# New Insights into the Immunological Changes in IL-10-Deficient Mice during the Course of Spontaneous Inflammation in the Gut Mucosa

**DOI:** 10.1155/2012/560817

**Published:** 2012-02-01

**Authors:** Ana Cristina Gomes-Santos, Thais Garcias Moreira, Archimedes Barbosa Castro-Junior, Bernardo Coelho Horta, Luisa Lemos, Deborah Nogueira Cruz, Mauro Andrade Freitas Guimarães, Denise Carmona Cara, Donna-Marie McCafferty, Ana Maria Caetano Faria

**Affiliations:** ^1^Departamento de Bioquímica e Imunologia, Instituto de Ciências Biológicas, Universidade Federal de Minas Gerais, Avenida Antônio Carlos 6627, 31270-901 Belo Horizonte, MG, Brazil; ^2^Departamento de Ciência de Alimentos, Faculdade de Farmácia, Universidade Federal de Minas Gerais, Belo Horizonte, MG, Brazil; ^3^Departamento de Morfologia, Instituto de Ciências Biológicas, Universidade Federal de Minas Gerais, Belo Horizonte, MG, Brazil; ^4^Gastrointestinal Research Group, Department of Physiology and Pharmacology, University of Calgary, 2500 University Dr. NW T2N 4N1, Calgary, AB, Canada

## Abstract

IL-10 is a regulatory cytokine that plays a major role in the homeostasis of the gut and this is illustrated by the fact that IL-10^−/−^ mice develop spontaneous colitis. In this study, IL-10^−/−^ mice were analyzed for immunological changes during colitis development. We found a reduced frequency of regulatory T cells CD4^+^CD25^+^Foxp3^+^ and higher frequency of activated T cells in the colon that precedes the macroscopic signs of the disease. Production of IL-17 and IFN-*γ* was higher in the colon. Colitis progression culminates with the reduction of CD4^+^LAP^+^ regulatory T cells in the intestine. Frequency of B1 cells and the secretory IgA production were both elevated. Despite these alterations, 16-week-old IL-10^−/−^ mice could be rendered tolerant by a continuous feeding protocol. Our study provides detailed analysis of changes that precede colitis and it also suggests that oral tolerance could be used to design novel alternative therapies for the disease.

## 1. Introduction

The intestine is the largest surface of contact between the body and the external environment [1]. Most contacts with foreign antigenic materials occur at the gut mucosa. It has been reported that 130–190 g of protein is absorbed in the small intestine daily [2] and the gastrointestinal tract harbours approximately 10^14^ microorganisms of more than 1000 species mostly in the colon [3]. All these antigenic contacts play an important role in the development of the immune system. Mice reared from weaning up to adulthood on a diet containing exclusively amino acids as nitrogen source have a drastic reduction in the gut-associated lymphoid tissue and in IgG/IgA production with an immunological phenotype that resembles suckling mice [4]. Germ-free mice display similar immunological alterations [5]. Under physiological conditions, the constant exposure to these natural antigens through the gut mucosa leads to local and systemic immunological activities, such as secretory immunoglobulin A (sIgA) production and oral tolerance induction [6].

 Oral tolerance has classically been defined as the specific suppression of cellular and/or humoral immune responses to an antigen previously given by the oral route [7]. Several mechanisms have been proposed for the development of oral tolerance, ranging from the deletion of antigen-specific T cells to immune deviation and suppression by regulatory T cells [6]. Studies in both mouse models and human tissues have suggested that inflammatory bowel disease (IBD) is a consequence of the breakdown of normal mucosal tolerance to luminal antigens. Chronic inflammatory bowel diseases are thought to arise from interacting genetic and environmental factors including altered T cell responses to intestinal antigens [8, 9]. Tolerance to autochthonous microbiota seems to be broken in individuals with IBD providing evidence that inflammatory reactivity to ubiquitous antigens from microbiota is implicated in the initiation and/or perpetuation of IBD [10].

Several murine models of colitis have highlighted the important role that abnormalities of the immune system, particularly those affecting T cells, may play in disease pathogenesis. These models include rats carrying the transgenes for HLA B27 and *β*2-microglobulin, and mice in which the genes coding IL-2, IL-10, and *α* or *β* chains of T cell receptors are absent [8]. In the same line, studies using T cell-restored immunodeficient mice have provided evidence that CD4^+^ T cells play a key role in the induction and regulation of intestinal inflammation. Cell transfer of CD45RB^high^ CD4^+^ T cells from normal mice donors into C.B-17 severe combined immunodeficient (SCID) mice led to the development of a severe inflammatory response in the colon [11]. The disease can be prevented by cotransfer of the CD45RB^low^ subset and interleukin IL-10 is an essential mediator produced by this regulatory T cell population [12].

IL-10 is produced by regulatory T cells and a variety of other cell types including epithelia, activated macrophages, dendritic cells, and B1 cells. IL-10 is a key immunosuppressive cytokine that acts directly on antigen-presenting cells (APC) to inhibit IL-12 secretion and down regulate the expression of MHC-II as well as costimulatory molecules such as CD80 and CD86 [13]. This modulatory action on APC indirectly inhibits T cell activation. Some studies have also suggested that IL-10 is a potent costimulant of B cell differentiation and immunoglobulin secretion [14]. The importance of this cytokine in shaping mucosal immune responses has been demonstrated by the spontaneous onset of gut inflammation in the IL-10-deficient (IL-10^−/−^) mouse [15].

Under conventional conditions, IL-10^−/−^ mice develop chronic enterocolitis by 2-3 months of age and there is no evidence of disease in neonates. The disease is characterized by weight loss, splenomegaly, and mild-to-moderate anemia. If maintained under specific pathogen-free (SPF) conditions, mice develop a limited form of colitis [16]. The typical inflammatory lesion found is discontinuous and transmural, affecting usually the lower gastrointestinal tract, with being the small intestine much less affected. Other pathological changes include epithelial hyperplasia, mucin depletion, crypt abscesses, ulcers, and thickening of bowel wall. The inflammatory infiltrate consists of lymphocytes, plasma cells, macrophages, eosinophils, and neutrophils [15]. Development of colitis in IL-10^−/−^ appears to be mediated by CD4^+^ T cells and an uncontrolled Th1 response [17]. In addition, the overproduction of numerous inflammatory mediators such as IL-1*β*, IL-6, tumor necrosis factor (TNF)-*α*, as found in cultures of mice with colitis [18].

In spite of the fact that enterocolitis in IL-10^−/−^ mice display features atypical of Crohn's disease including marked crypt hyperplasia, the rare occurrence of granulomas, fibrosis, and the absence of fissures and fistulae, this experimental model resembles Crohn's disease in the transmural and discontinuous inflammation. This inflammation can affect not only the colon but also the small intestine [19]. In fact, Crohn's disease in humans may affect any segment of the digestive tract but most frequently involves the small intestine [20].

This study aimed to characterize morphological and immunological alterations in gut mucosa of IL-10^−/−^ at 6, 10, or 16 weeks of ages, covering a period since the onset of symptoms until established enterocolitis. We analysed the inflammatory features of each stage of the disease as well as putative changes in immunoregulatory mechanisms that may be involved. Since most of genetically deficient animals used as experimental models for IBD are born without colitis signs [21, 22], they constitute an opportunity to study the immunological changes preceding the disease.

## 2. Material and Methods

### 2.1. Animals

Wild-type (WT) and IL-10-deficient (IL-10^−/−^) mice on a 129Sv/Ev background were obtained from Donna-Marie McCafferty's laboratory (Calgary University, Calgary, Canada). All mice were bred and housed in our facility at Universidade Federal de Minas Gerais, Brazil. Mice were kept in microisolators with autoclaved standard chow and water until weaning. After weaning, all mice were maintained in a conventional facility (open cages). Animals were studied at 6, 10, and 16 weeks of age and age-matched wild-type 129Sv/Ev mice were used as controls. All procedures were approved by the local ethical committee for animal research (Protocol no. 170/2008, CETEA-UFMG, Brazil).

### 2.2. Macroscopic and Microscopic Assessment of Colitis

The colon was excised and colonic inflammation assessed using a previously defined scoringsystem, which includes features of clinical colitis, such as the presence or absence of adhesions, strictures and diarrhea (diarrhea was defined as loose, watery stool), and the bowel wall thickness (measured in mm). Samples of colon were fixed in formalin and processed for microscopic analysis. Hematoxylin-eosin-stained sections were blindly scored based on a semiquantitative scoring system described previously [23] where the following features were graded: extent of destruction of normal mucosal architecture (0: normal; 1, 2, and 3: mild, moderate, and extensive damage, resp.), presence and degree of cellular infiltration (0: normal; 1, 2, and 3: mild, moderate, and transmural infiltration, resp.), extent of muscle thickening (0: normal; 1, 2 and 3: mild, moderate and extensive thickening, resp.), presence or absence of crypt abscesses (0: absent; 1: present) and the presence or absence of goblet cell depletion (0: absent; 1: present). Scores for each feature were summed up to a maximum possible score of 11.

### 2.3. Intestinal Tissue Preparation and Cytokine Assay

The intestine was separated into duodenum, proximal jejunum, distal jejunum, ileum, and colon and placed in buffer solution (1 mL/0.1 g). Tissue fragments were homogenized and centrifuged for 10 minutes 600 g at 4°C. Supernatants were collected for cytokine assay. Plates were coated with purified monoclonal antibodies reactive with cytokines IL-17A, IL-6, TGF-*β*, and IFN-*γ* (BD-Pharmingen) overnight at 4°C. In the, following day, wells were washed, supernatants were added, and plate was incubated overnight at 4°C. In the third day, biotinylated monoclonal antibodies against cytokines were added and plates were incubated for 2 hours at room temperature. Color reaction was developed at room temperature with 100 *μ*L/well of orthophenylenediamine (1 mg/mL), 0.04% H_2_O_2_ substrate in sodium citrate buffer. Reaction was interrupted by the addition of 20 *μ*L/well of 2 N H_2_SO_4_. Absorbance was measured at 492 nm by ELISA reader (BIO-RAD).

### 2.4. Cell Preparation and Flow Cytometry Analysis

Intraepithelial lymphocytes (IELs) were isolated by a modified version of the method described by Davies and Parrott [24]. Briefly, the entire length of small and large intestine were dissected, opened longitudinally, washed with PBS, and cut into small pieces. Tissue fragments were placed in Petri dishes and washed three times in calcium and magnesium-free HBSS containing 2% fetal bovine serum (FBS). After that, tissue fragments were transferred to culture flasks and incubated at 37°C in HBSS containing 1 mM DL-dithiothreitol (DTT-Sigma) for 30 min, twice. Supernatants were filtered through a 70 *μ*m cell strainer and the IEL fraction kept on ice. For *lamina propria* (LP) cell isolation, tissue fragments were incubated with 100 U/mL of collagenase II (Sigma) for 60 min at 37°C in a shaker. Supernatants were passed through a 70 *μ*m cell strainer and then ressuspended in medium. Cells were suspended in 44% Percoll solution, which was layered on top of 67% Percoll solution and centrifuged at 600 g for 20 min at 4°C. IELs were collected from the interface between the Percoll gradients. Cells from IEL and LP compartments were then labelled with FITC-conjugated anti-mouse CD4, CD5 and CD25, PE-conjugated anti-mouse CD25, Thy1.2, CD44, TCR*γδ*, TCR*αβ* and Foxp3, Cy5-conjugated anti-mouse CD69, CD19, and Biotin anti-mouse LAP (BD Pharmingen). Cells from spleen, Peyer's patches, peritoneum, and bone marrow were labelled with FITC-conjugated anti-mouse CD5 and Cy5-conjugated anti-mouse CD19. Cells were analyzed by a FACSCan (Becton & Dickinson) and data were analyzed by FlowJo (TreeStar). At least 30,000.00 events were counted for each sample.

### 2.5. Analysis of Ig Isotypes by ELISA

Levels of Ovalbumin-(Ova-) specific and total immunoglobulins were determined by ELISA. Briefly, 96-well plates (Nunc) were coated with 2 *μ*g/well Ova or 0.1 *μ*g goat anti-mouse UNLB antibody, in coating buffer pH 9.8 overnight. Wells were washed and blocked with 200 *μ*L of PBS contain 0.25% casein for 1 h at room temperature. Sera were added to the plate and incubated for 1 h at room temperature, plates were washed, then peroxidase-streptavidin goat anti-mouse or rat anti-goat (Southern Biotechnology) 1 : 15000 was added, and plates were incubated for 1 h at 37°C. Color reaction was developed at room temperature with 100 *μ*L/well of orthophenylenediamine (1 mg/mL) (Sigma), 0.04% H_2_O_2_ substrate in sodium citrate buffer. Reaction was interrupted by the addition of 20 *μ*L/well of 2 N H_2_SO_4_. Absorbance was measured at 492 nm by an ELISA microplate reader (Bio-Rad).

### 2.6. Oral Tolerance Induction

Oral tolerance to Ova was induced by intragastric administration (gavage) of a single dose of 20 mg Ova (Sigma) in 0.2 mL saline (0.15 M NaCl), 7 days before primary immunization. The control group received 0.2 mL of saline. Alternatively, mice received 4 mg/mL solution of Ova in water as their exclusive drinking fluid. The average voluntary intake of a mouse was about 5 mL in 24 h; therefore, the animals were presumed to ingest 20 mg Ova/day. Control groups received filtered tap water. Oral treatment was discontinued 7 days before parenteral immunization. Mice were actively sensitized by an intraperitoneal injection of 0.2 mL of saline containing 10 *μ*g of Ova adsorbed in 1 mg of aluminum hydroxide. Fourteen days later, the animals received the same dose of Ova in PBS.

### 2.7. Statistical Analysis

Results were expressed as the mean ± standard error of the mean (SEM). The Kolmogorov-Smirnov test, confirmed normal distribution of samples. Significance of differences among groups was determined by Student's *t*-test or analysis of variance (ANOVA) (Tukey's posttest). Means were considered statistically different when *P* < 0.05.

## 3. Results

### 3.1. Assessment of Colitis during the Course of Intestinal Inflammation

**Figure 1:**
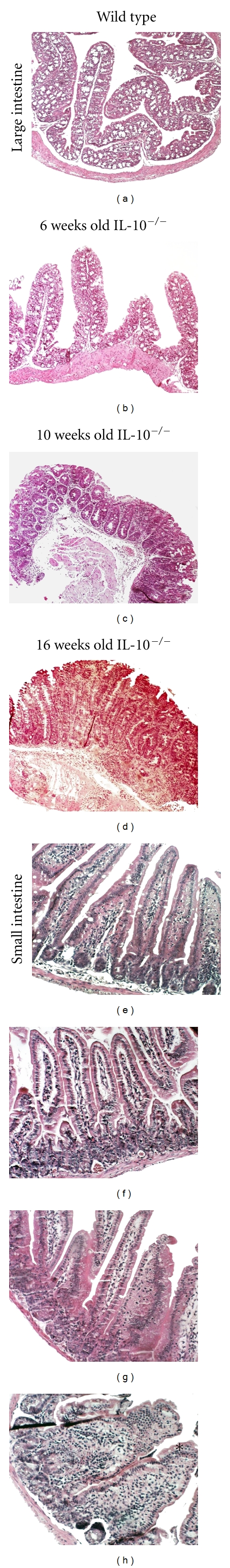
Histopathology of intestinal changes in IL-10^−/−^ mice. (a) Histology of proximal colon representative of wild-type (129Sv/Ev) mice (40x). No cellular infiltration between glands was found. (b) Proximal colon of 6-week-old IL-10^−/−^ mice (40x). Mucosa and submucosa are similar to the ones in wild-type mice. (c) Proximal colon of 10-week-old IL-10^−/−^ mice (40x). Multifocal infiltrations of leucocytes in the mucosa and submucosa. (d) Proximal colon of 16-week-old IL-10^−/−^ mice (40x). Presence of transmural inflammation affecting the muscular layer can be observed. (e) Proximal jejunum representative of wild-type mice (100x). (f) Proximal jejunum of 6-week-old IL-10^−/−^ mice (100x). (g) Proximal jejunum of 10-week-old IL-10^−/−^ mice (100x). (h) Proximal jejunum of 16-week-old IL-10^−/−^ mice showing enteritis and cellular infiltration in the *lamina propria* (asterisks) (100x). Tissues were stained with hematoxylin and eosin.

**Figure 2:**
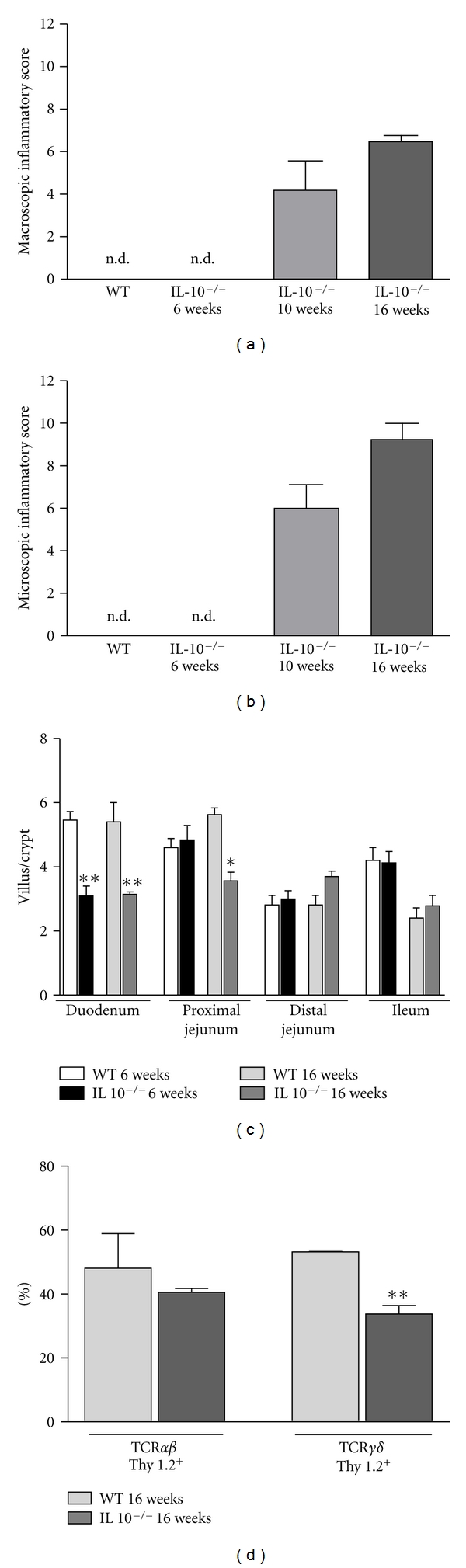
Inflammatory score of IL-10^−/−^ and IEL profile. (a) Macroscopic score of IL-10^−/−^ mice at 6, 10, or 16 weeks of age and control age-matched wild-type mice. (b) Microscopic score of IL-10^−/−^ mice at 6, 10, or 16 weeks of age and control age-matched wild-type mice obtained by histological analysis of the colonic tissues. (c) Villus/crypt ratio of duodenum, proximal jejunum, distal jejunum, and ileum of IL-10^−/−^ mice at 6, or 16 weeks of age. Bars represent the mean ± SEM of 5 mice per group. Asterisks represent differences from age matched WT groups in the same intestinal segment (*P < 0.05 or **P < 0.01). (d) Flow cytometry analysis with frequency of IEL isolated from 16-week-old IL-10^−/−^ and control mice stained with fluorescent antibodies to *αβ*TCR, *γδ*TCR, and Thy1.2. Cells were analyzed inside the total lymphocytes gate. Bars represent the mean ± SEM of 3 mice per group. Significant difference (**P < 0.01) is indicated by the asterisk. n.d means non detected.

Macroscopic and histology scores of colonic mucosa were observed in 6-, 10-, or 16-week-old IL-10^−/−^ mice and age-matched wild-type mice. IL-10^−/−^ mice displayed normal colonic histological appearance at 6 weeks of age without macroscopic signs of disease (Figures 1(b), 2(a), and 2(b)). By 10 weeks of age, IL-10^−/−^ mice developed a mild colitis (Figure 1(c)), the severity of which reached a plateau at 16 weeks of age (Figure 1(d)). The macroscopic score did not increase after disease was established since 10- or 16-week-old IL-10^−/−^ mice showed similar scores (Figure 2(a)).

### 3.2. Morphology of Small Intestine and the IEL Profile in IL-10-Deficient Mice

Regarding the impact of inflammatory changes in small intestine, a previous study has described that IL-10^−/−^ mice can develop enteritis in conventional conditions [15]. Histological analysis of the small intestine revealed signals of inflammation only in the proximal jejunum of 16-week-old IL-10^−/−^ mice (Figure 1(h)). They had altered villus/crypt ratio (Figure 2(c)) due to villus shortening. Noteworthy, 6-weeks-old mice presented villus/crypt ratio similar to older mice (Figure 2(c)) but this was due to higher crypt size. Intraephithelial lymphocytes (IELs) are involved in maintenance of intestinal epithelial cell integrity [25]. Frequencies of TCR*αβ* and TCR*γδ* IEL populations were analyzed in 16-week-old IL-10^−/−^ mice. We used Thy1.2 as a marker to discriminate activated IELs. Despite the similar frequencies of TCR*αβ* and TCR*γδ* IELs, there was a decrease in the percentage of Thy1.2^+^TCR*γδ* ^+^ in the IEL population in IL-10^−/−^ mice, whereas no change was found in the frequency of Thy1.2^+^TCR*αβ* ^+^ IELs (Figure 2(d)).

### 3.3. IL-10-Deficient Mice Had a Reduced Frequency of Regulatory T Cells in the Small Intestine

**Figure 3:**
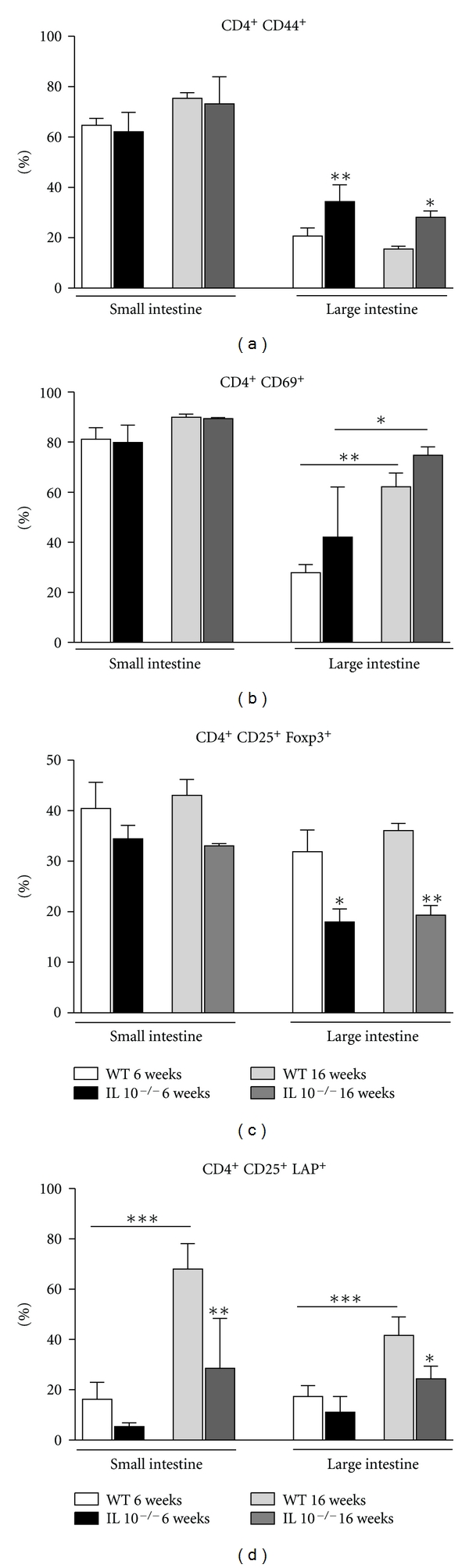
T lymphocyte profile in the *lamina propria* of small and large intestine of IL-10^−/−^ mice during the course of intestinal inflammation. *Lamina propria* T cells from small and large intestine were obtained from either 6- or 16-week-old IL-10^−/−^ mice. Age-matched 129Sv/Ev (WT) mice were used as controls. Frequencies of CD4^+^CD44^+^ (a), CD4^+^CD69^+^ (b), CD4^+^CD25^+^Foxp3^+^ (c), and CD4^+^CD25^+^LAP^+^ (d) T cells gated in CD4^+^ T cells were assessed by flow cytometry. Bars represent the mean ± SEM of 4 mice per group. Significant differences (*P < 0.05, **P < 0.01 or ***P < 0.001) are indicated by asterisks.

In agreement with other studies, we found an increase in the frequency of CD4^+^CD44^+^ in the colon of IL-10-deficient mice at 6 and 16 weeks of age but a nonaltered frequency in the small intestine (Figure 3(a)). There was no difference in the frequency of T cells expressing the early activation marker CD69^+^ in the small and large intestine segments of either 6- or 16-week-old IL-10^−/−^ mice when compared to their wild-type counterparts. Of note, frequency of activated CD69^+^ T lymphocyte population increased with age in both wild-type and IL-10^−/−^ mice but only in the large intestine (Figure 3(b)). CD4^+^CD25^+^Foxp3^+^ T cells were reduced in the *lamina propria* of large intestine of IL-10^−/−^ mice regardless of their age (Figure 3(c)). The CD4^+^CD25^+^LAP^+^ population of lymphocytes was reduced in the small and large intestines of IL-10^−/−^ at 16 weeks of age. However, the frequency of CD4^+^CD25^+^LAP^+^ T cells was enhanced with age in wild-type mice in both small and large intestines (Figure 3(d)).

### 3.4. Changes in Cytokine Secretion in Small and Large Intestines of IL-10-Deficient Mice

**Figure 4:**
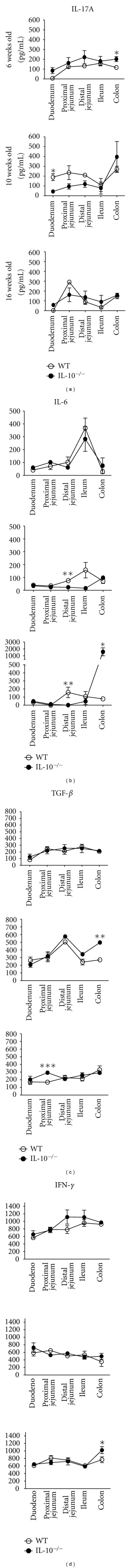
Production of cytokines in the intestinal mucosa during the course of intestinal inflammation. Intestines from IL-10^−/−^ at 6, 10 or 16 weeks of age were removed, separated into duodenum, proximal jejunum, distal jejunum, ileum and homogenized in extract buffer. Age-matched 129Sv/Ev (WT) mice were used as controls. Extract supernatant was collected for cytokine assay. IL-17A (a), IL-6 (b), TGF-*β* (c), and IFN-*γ* (d) were measured by ELISA. *n* = 5 mice per group. Asterisks represent differences among groups in the same intestinal segment (*P < 0.05, **P < 0.01 or ***P < 0.001).

The cytokine profile in IL-10^−/−^ mice was well characterized in colonic extract [18]. However, no information is available about the effect of the lack of IL-10 in the small intestine. IL-17A production enhanced in the colon of 6-week-old IL-10-deficient mice and diminished in the duodenum of 10-week-old IL-10^−/−^ mice (Figure 4(a)). We found alterations in IL-6 in the distal jejunum of IL-10^−/−^ mice with 10 and 16 weeks of age. Levels of IL-6 raised in the colon of 16-week-old IL-10^−/−^ mice (Figure 4(b)). On other hand, production of the anti-inflammatory cytokine TGF-*β* in IL-10^−/−^ mice increased in the colon at 10 weeks of age and in proximal jejunum at 16 weeks of age (Figure 4(c)). 16-week-old IL-10^−/−^ mice had increased levels of IFN-*γ* in the colon (Figure 4(d)).

### 3.5. Frequency of B1 Lymphocytes Increased in Peritoneum and Intestinal lamina propria of IL-10-Deficient Mice with Colitis

**Figure 5:**
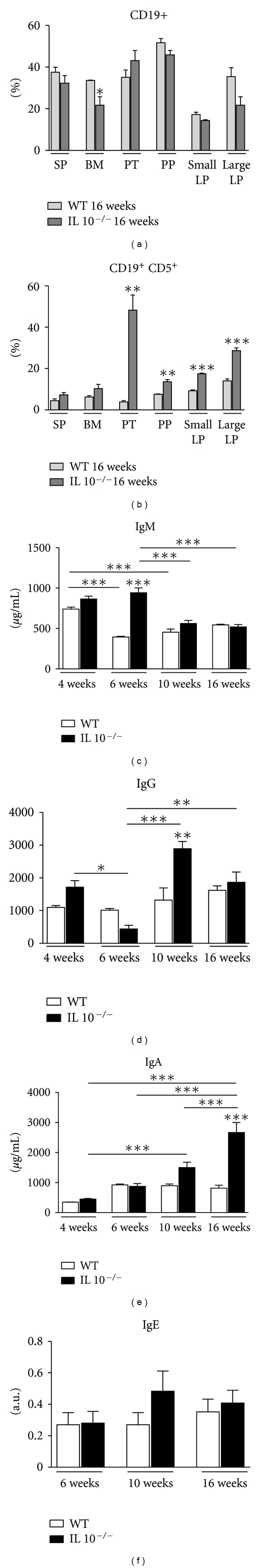
B cells and immunoglobulins isotype during the development of enterocolitis. Cells from spleen (SP), bone marrow (BM), Peritoneum (PT), Peyer patches (PP), or *lamina propria* (LP) from small or large intestine were obtained from 16-week-old IL-10^−/−^ mice. Frequencies of CD19^+^ B cells (a) or CD19^+^CD5^+^ (b) gated on lymphocytes or CD19^+^ population, respectively, were assessed by flow cytometry. Bars represent the mean ± SEM of 4 mice per group. Asterisks represent difference between groups (*P* < 0.05). Sera from 4-, 6-, 10-, or 16-week-old IL-10^−/−^ mice were collected and total IgM (c), IgG (d), IgA (e), and IgE (f) were measured by ELISA. A.U.: arbitrary units. Bars represent the mean ± SEM of 5 mice per group. Significant differences (*P < 0.05, **P < 0.01 or ***P < 0.001) are indicated by asterisks.

Since IL-10 is a potent costimulant of B cell differentiation, immunoglobulin secretion and generation of B1 cells [14], our next step was to investigate the impact of IL-10 deficiency in B lymphocyte populations and immunoglobulin production. There was no significant change in the frequency of B lymphocytes in spleen (SP), peritoneum (PT), Peyer's patches (PP), and gut *lamina propria* (LP) but frequency of these cells was diminished in bone marrow (BM) (Figure 5(a)). Interestingly, frequency of B1 lymphocytes in peritoneum, Peyer's patches, and *lamina propria* of both small and large intestine was augmented in IL-10^−/−^ mice at 16 weeks of age (Figure 5(b)).

### 3.6. Serum Levels of Immunoglobulins Were Altered during the Course of Disease in IL-10-Deficient Mice

We investigated whether production of the different classes of immunoglobulin was altered in IL-10-deficient mice since weaning and during the course of gut inflammation. There was no difference in the serum levels of IgG, IgM, and IgA in IL-10-deficient mice at 4 weeks of age (Figures 5(c)–5(e)). However, at 6 weeks of age, IL-10-deficient mice had enhanced IgM levels and reduced levels of IgG when compared to levels found in mice at 4 weeks of age. IgA levels were unchanged at this age (Figures 5(c)–5(e)). At 10 weeks of age, IL-10^−/−^ mice had higher concentration of serum IgG. Serum IgA enhanced in IL-10^−/−^ mice from 4 to 10 weeks of age and rose again in 16 week-old mice (Figure 5(e)). Levels of IgE were similar in all ages analysed (Figure 5(f)).

### 3.7. IL-10-Deficient Mice with Established Gut Inflammation Can Be Rendered Tolerant Only by Continuous Feeding

**Figure 6:**
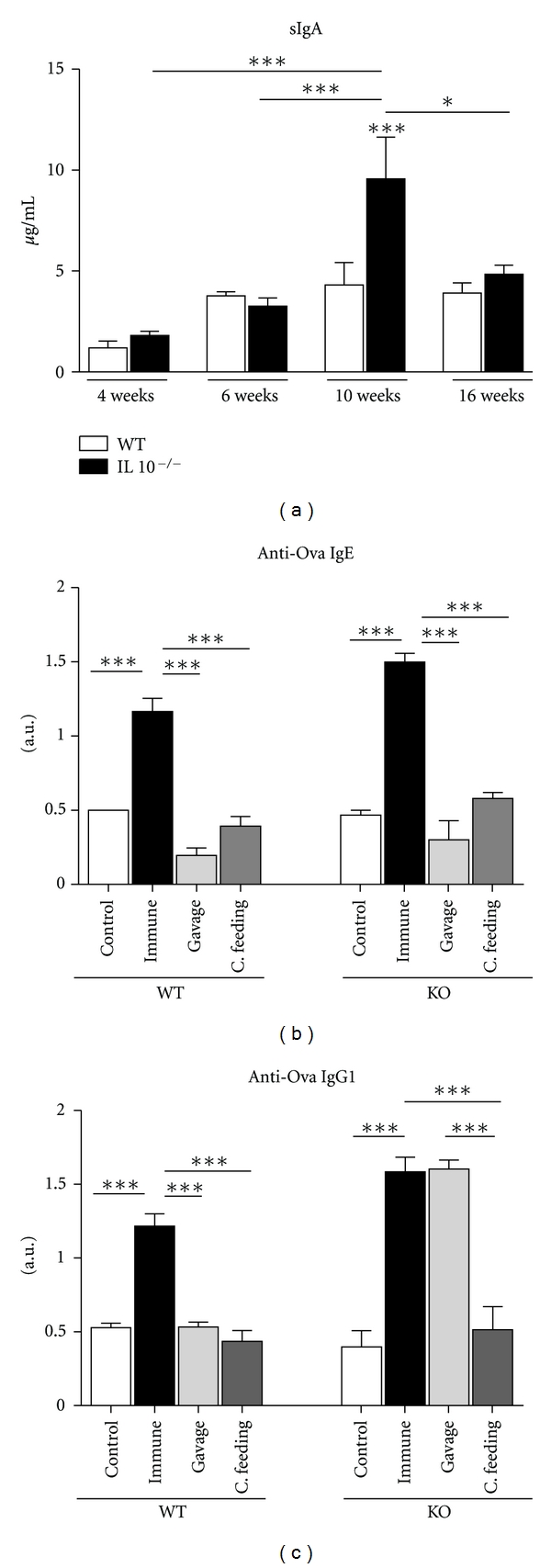
Secretory IgA and oral tolerance. Intestinal feces from 4-, 6-, 10-, or 16-week-old IL-10^−/−^ mice were collected and total sIgA (a) was measured by ELISA. 16-week-old IL-10^−/−^ or wild-type mice received Ova either by gavage or by continuous feeding. Seven days later mice were sensitized by an intraperitoneal (i.p.) injection of 0.2 mL saline (0.9% NaCl) solution containing 10 *μ*g OVA (Sigma, St. Louis, MO) adsorbed in 1 mg aluminum hydroxide. Fourteen days later, animals received the same dose of OVA in PBS. Control animals received 0.2 mL sterile saline. Anti-Ova IgE and IgG1 antibodies were measured by ELISA. A.U.: arbitrary units. Bars represent the mean ± SEM of 5 mice per group. Asterisks represent difference between groups (*P < 0.05 or ***P < 0.001).

Finally, we tested the impact of IL-10 deficiency and colitis development in the two major immunological activities that take place at the gut mucosa: secretory IgA (sIgA) production and oral tolerance induction. Concentration of sIgA was measured in the feces. Similarly to what has already been described in the literature, IL-10^−/−^ mice had enhanced levels of sIgA at the time of established inflammation (10 weeks of age) but no difference was found in mice before the onset of colitis at 4 or 6 weeks of age. Interestingly, mice at 16 weeks of age with overt inflammation did not show alterations in sIgA again (Figure 6(a)). To test for oral tolerance induction, mice were either fed 20 mg Ova by intragastric administration (gavage) or given an Ova solution as the only liquid source for one day (continuous feeding). They were intraperitoneally immunized with the same antigen in adjuvant 7 days thereafter. Levels of anti-Ova IgE were reduced in both wild-type (129Sv/Ev) and in IL-10^−/−^ mice that received Ova by either continuous feeding or gavage before immunization when compared to the immunized group (Figure 6(b)). However, only IL-10-deficient mice that were continuously fed Ova could be rendered tolerant for anti-Ova IgG1 production (Figure 6(c)).

## 4. Discussion

Mouse models of intestinal inflammation have played a key role in understanding the mechanisms that govern the inflammatory response in the intestine, and in designing new therapeutic strategies for human Crohn's disease and ulcerative colitis. Experimental models of IBD usually involve defects in epithelial integrity/permeability or in regulatory elements of the immune system [8]. Several pathological and immunological changes are significantly different between these models. For example, the chemical model induced by dextran sulfate sodium (DSS) display inflammatory features similar to ulcerative colitis, while in IL-10^−/−^ the inflammation resembles Crohn's disease featuring transmural inflammation in colonic mucosa. Indeed, we found that in the IL-10^−/−^ mice there was an overproduction of IFN-*γ* and IL-17 (Figure 4), whereas in DSS-induced model of colitis occurred a prevalence of IL-6, TNF-*α*, and INF-*γ* without alterations in IL-17 production (data not shown). Of note, we observed reduced frequencies of regulatory T cells in mice with DSS-induced colitis (data not shown) similarly to what was found in IL-10^−/−^ mice (Figure 3). However, in DSS-induced colitis, the earliest histological change that predated clinical colitis was the loss of crypt epithelial cells. Inflammation became significant only after the appearance of erosions. On the other hand, in IL-10-deficient mice, inflammation precedes the establishment of clinically detected disease and occurred spontaneously. Therefore, the model of IL-10^−/−^ mouse provides an opportunity to study pathophysiology and immunological changes that precede and accompany the development of the disease.

In this study we used IL-10-deficient mice in the 129Sv/Ev genetic background. Although macroscopic disease can be detected at 3 months of age in IL-10-deficient mice, severity of intestinal lesions vary with the genetic background of the mice. They are most severe in 129Sv/Ev and BALB/c strains, of intermediate severity in 129 x C57BL/6J hybrid mice, and least severe in the C57BL/6J strain. Thus, the 129Sv/Ev strain is considered the one with highest susceptibility to colitis development with homogenous clinical manifestations of the disease among susceptible animals [19]. Since in SPF conditions these mice exhibited only small lesions in the mucosa of proximal colon [16], we chose to undertake our study with mice maintained under conventional conditions, which lead to the full development of enterocolitis.

At 6 weeks of age, just after weaning, no histological sign of inflammation was observed in the large intestine of these mice. On the other hand, IL-10-deficient mice at 10 weeks of age showed already established enterocolitis with infiltration of leucocytes in the gut mucosa and submucosa areas. Severity of the disease progressed as they aged, and 16-week-old mice showed a transmural inflammation in the colon. Parameter such as onset and severity of enterocolitis in IL-10^−/−^ mice varies according to the animal facility that mice are kept. Rederivation of IL-10-deficient mice from conventional SPF environments into germ-free isolators eliminates colitis development, clearly demonstrating the influence of microbiota in the establishment of disease [16]. In addition, IL-10-deficient C3H mice from the same parenteral breeding stocks but maintained in two different facilities had significant differences in histopathological scores at the same age. These differences were attributed to the source of diet and its ingredients and to the water treatment (autoclaved or not) because health monitoring of the two colonies indicates the same SPF status [26]. Berg and coworkers reported small multifocal infiltrates, in *lamina propria* of colon in 3-week-old IL-10^−/−^ mice whereas 12-week-old IL-10^−/−^ mice had multifocal lesions and epithelial hyperplasia in all regions of large intestine [19]. Thus, in mice housed in our animal facility, the onset of enterocolitis was delayed but the progression was faster.

In this study, we evaluated not only inflammatory changes in the large intestine but also in the small intestine of IL-10-deficient mice. Although there was no visible inflammation in the small intestines at 6 weeks of age, the villus/crypt ratio was reduced and this alteration was observed until adulthood (16 weeks of age). Altered villus/crypt morphology has been reported in a number of immune-mediated bowel disorders, including celiac disease, Crohn's disease, and ulcerative colitis. This type of change is due to accelerated epithelial turnover and apoptosis triggered by cytokines released from infiltrating inflammatory cells and from enterocytes themselves [27]. This could contribute to the inflammatory enteritis that we found in 16-week-old IL-10^−/−^ mice.

Small intestinal mucosa harbours a particular population of lymphocytes named intraepithelial lymphocytes (IELs). IELs are in the first line of mucosal surface intertwined with epithelial cells, and the main subpopulations of IELs, TCR*αβ* ^+^ and TCR*γδ* ^+^, are both described as involved in inhibition of cytotoxic T cells (CTL). The intraepithelial lymphocyte compartment probably provides a first line of defense against infectious pathogens attacking the surfaces of the body and also provides a link between innate and acquired immunity [25, 28]. TCR*γδ* ^+^ IELs are also related to immunoregulatory roles such as maintenance of epithelial tissue integrity [25], oral tolerance induction [29], and colitis modulation [30]. In acute colitis induced by administration of either 2,4,6-trinitrobenzene sulfonic acid (TNBS) or DSS, a protective role of *γδ* T cells has been demonstrated [30]. Our group has shown recently that the frequency of *γδ* ^+^ IELs is diminished in aged mice and that this aging-associated change parallels a reduction in susceptibility to oral tolerance induction [31]. In this study, we observed that the frequency of TCR*αβ* ^+^ and TCR*γδ* ^+^ T lymphocytes in the IEL compartment did not change (data not shown), but there was a reduction in the frequency of activated TCR*γδ* ^+^ Thy1.2^+^ cells in IL-10-deficient mice with established inflammation. Since TCR*γδ* ^+^ IELs are involved in regulatory activities in the gut mucosa, this reduction can represent an instance of immunoregulatory failure associated with disease development in IL-10-deficient mice.

Classical studies reported that the enterocolitis in IL-10-deficient mice is associated with uncontrolled cytokine production by activated macrophages and CD4^+^ Th1-like T cells [19]. The excessive generation of IFN-*γ*-producing T cells (Th1) driven by IL-12 produced by antigen-presenting cells was described to be responsible for the initiation of disease [32]. However, later studies revealed that IL-23, but not IL-12, drives the intestinal inflammation in IL-10^−/−^ mice. A critical target of IL-23 is memory T cells, which produce the proinflammatory mediators IL-17 and IL-6 [33]. We showed that levels of IFN-*γ* were elevated in the colon of 16-week-old but not of young IL-10^−/−^ mice. Moreover, 6-week-old IL-10^−/−^ mice had increased levels of IL-17A in their colonic mucosa. These results are consistent with the findings that IL-17A is the cytokine that initiate the intestinal inflammation in IL-10^−/−^ mice followed by an enhanced production of IFN-*γ*. Th17 cells can convert to Th1 cells through IL-17 induction of IL-12 and IL-23 production by dendritic cells at colonic mucosa [34]. Recently, Mikami and coworkers showed that cotransfer of the mixed Th1/Th17 CD4^+^ T cells from IL-10^−/−^ with colitis with Th1 CD4^+^ T cells from CD4^+^CD45^high^-induced RAG^−/−^ mice with colitis into RAG^−/−^ mice ameliorate wasting disease. Thus, it seems that Th17 cells compete with Th1 cells, and that predominant secretion of IFN-*γ* is related to more severe colitis [35]. Moreover, macrophages are usually activated by IFN-*γ* to produce IL-6 and this could explain the concomitant increase in IFN-*γ* and IL-6 levels in the colon of 16-week-old IL-10^−/−^ mice [36]. In light of these results, TGF-*β* production in the colon of 10-week-old IL-10^−/−^ mice may represent an attempt to regulate inflammation by the direct regulatory action of TGF-*β*. Indeed, production of IL-6 in the colon of 10-week-old IL-10^−/−^ was not high. It is also remarkable that the shift from IL-17A into IFN-*γ* production in the colonic mucosa of IL-10^−/−^ mice coincided with more severe colitis, spreading of inflammation to the small intestine (Figure 1) and deterioration in the clinical status of the animals.

Cytokine profile was also evaluated in all segments of the small intestine. We found enhanced levels of IL-17A in the duodenum of 10-week-old IL-10^−/−^ mice. On the other hand, there were higher levels of TGF-*β* in the colon at the same time point. At 16 weeks of age, TGF-*β* was also produced in the proximal jejunum. Interestingly, the enhanced IL-17A production observed at 10 weeks of age disappeared at 16 weeks of age (Figure 4(b)). The detected upregulation of TGF-*β* in 10-week-old mice could be an immunoregulatory process triggered in the small intestine to control inflammation. However, it was not a successful modulatory event since histological analysis of the small intestine at that stage showed that inflammation was pronounced in the colon and also in the small intestine. This suggests that although TGF-*β* can be an important compensatory mechanism in the immune homeostasis of IL-10-deficient mice, these animals show no other inflammatory disorder. Nevertheless, in the gut mucosa, IL-10 seems to be critical and overproduction of TGF-*β* was not enough to control intestinal inflammation.

Despite the absent intestinal inflammation in young mice, we found enhanced frequency of CD4^+^ T cells expressing a memory phenotype (CD44^high^) in the large intestinal mucosa before the establishment of colitis (6 weeks of age). Berg and coworkers showed that increased numbers of T cells were present in IL-10-deficient mice already at 3 weeks of age [19]. In a study using the G*α*i2-deficient mouse model of ulcerative colitis, increased numbers of mucosal lymphocytes expressing the CD44^high^ marker were isolated from mice before the onset of colitis [21]. Presumably, the T cell influx into the colonic *lamina propria* is an important factor in the subsequent establishment of colitis. In IL-10-deficient mice with severe disease, the high frequency of CD44^high^ was maintained. We had distinct results for T lymphocytes expressing the early activation marker CD69^+^. There was an enhanced frequency of CD69^+^ in both 129Sv/Ev wild-type and IL-10^−/−^ mice from 6 weeks to 16 weeks of age. This indicates that an age-related increase in activated CD4^+^ lymphocytes that is consistent with other studies [31] and that can be explained by the continuous exposure to antigens under conventional housing. This seemed to be associated with microbiota stimulation since there is no equivalent change in the frequency of activated CD4^+^ T cells in the small intestine where bacterial colonization is much less intense.

Induced CD4^+^CD25^+^Foxp3^+^ T cells have already been shown as important players in the homeostasis of gut mucosa [37]. A reduced frequency of CD4^+^CD25^+^Foxp3^+^ regulatory T cells was found in the large intestine of mice as young as 6 weeks old. This could be directly related to the IL-10 deficiency. Moreover, IL-10 secreted by CD11b^+^CD11c^+^ dendritic cells is a fundamental cytokine for the maintenance of Foxp3 expression in induced regulatory T cells in the periphery during colitis development [38].

Our next step was to analyze the frequency of CD4^+^LAP^+^ T cells during colitis development in these mice. CD4^+^LAP^+^ T cells represent a subset of regulatory T cells expressing TGF-*β* bound to their membranes in its precursor form (associated with the latent associated peptide, LAP). CD4^+^CD25^+^LAP^+^ T cells have been shown to participate in the control of intestinal inflammation in experimental models of colitis [39] and CD4^+^LAP^+^ T cells, either expressing CD25 or not, have recently been reported as a distinct subset of T cells [40]. Frequency of CD4^+^CD25^+^LAP^+^ was not affected in young IL-10-deficient mice. However CD4^+^CD25^+^LAP^+^ T cells were decreased in IL-10-deficient mice at 16 weeks of age both in the colon and in the small intestine. The decrease in frequency of CD4^+^CD25^+^LAP^+^ T cells was observed in the small intestine of 16-week-old IL-10^−/−^ in the same time point of TGF-*β* augment in proximal jejunum. Thus, it is likely that other cell types, including macrophages and the gut epithelial cells, were responsible for the production of TGF-*β* and not CD4^+^LAP^+^ T cells. We also found enhanced frequencies of CD4^+^LAP^+^ T cells in wild-type mice at 16 weeks of age when compared to the frequencies found in 6-week-old mice. Our group described similar data in a recent study on age-related alterations in the gut mucosa. An increase in CD4^+^CD25^+^LAP^+^ cells was observed in mesenteric lymph nodes and Peyer's patches of 6- to 24-month-old mice when compared to young animals (2-month-old), suggesting that these T cell subsets augment after sexual maturity but remain stable afterwards [31].

Not only the cell-mediated but the humoral components of the immune system have been implicated in the pathogenesis of human and mouse models of IBD. Anticolon antibodies have been detected in IL-2^−/−^ mice [41]. Antibody reactive with enteric microbiota such as *Campylobacter jejuni*, have been identified in human patients, particularly those with ulcerative colitis [42]. In this study, we find enhanced levels of serum IgG and IgA in IL-10-deficient mice with established and severe colitis, respectively. Our findings are in agreement with other studies on IL-10-deficient mice. Kuhn and colleagues were the first authors to demonstrate elevated levels of serum IgG1 and IgA in 8-week-old IL-10^−/−^ mice [15]. Davidson and coworkers also showed the cross-reactivity of IL-10^−/−^ mice serum Ig with colon epithelial and nonepithelial cells in the majority of sera tested. However, the same authors created the B cell-deficient (B^−/−^) strain of lL-10^−/−^ mice and this mice acquired a severe colitis analogous to that of lL-10^−/−^ mice, implying that B cells were not the primary mediator of IBD in this model [17]. Therefore, we cannot say whether that these alterations were a compensatory response to the inflammation or that they were simply a phenomenon related to inflammation of the colon. IL-10-deficient mice at the age of 4–6 weeks had a similar percentage of B cells in thymus and spleen and normal B1 cell subset in the peritoneum [15]. B1 cells were originally identified as CD5^+^ B cells [43]. Surprisingly, frequency of CD19^+^CD5^+^ cells was increased in the *lamina propria* of small and large intestine as well as in peritoneum and Peyer's patches of 16-week-old IL-10^−/−^ mice. B1 cells from the peritoneum produce predominantly IgM, but class switch to IgA has been shown to occur in the B cell population [44] and *lamina propria* B1 cells are responsible for at least half of the secretory IgA in the gut mucosa [45]. Serum IgA levels enhanced concomitantly with inflammation in IL-10^−/−^ mice. Secretory IgA levels were also higher at 10-week-old mice. Thus, it is reasonable to speculate that B1 cells are the main source of this increment of IgA.

Oral tolerance is a major and common consequence of oral administration of antigen [6]. Although several mechanisms have been proposed to explain the induction, production of anti-inflammatory cytokines such as TGF-*β* and IL-10 and induction of regulatory T cells such as CD4^+^CD25^+^Foxp3^+^ and CD4^+^LAP^+^ T cells seem to be critical for oral tolerance induction [46]. There were previous and controversial reports on the role of IL-10 in this process. Rizzo and coworkers showed that mice genetically engineered to lack IL-4, IL-10 or both cytokines were refractory to oral tolerance [47]. Later, Aroeira and coworkers showed that in vivo depletion of IL-10 with monoclonal antibodies did not affect oral tolerance induction in mice [48]. In the present study, we observed that oral tolerance to IgE could be induced in 16-week-old IL-10-deficient mice by the two regimens of feeding used, gavage, and continuous feeding. However, only continuous feeding of the antigen, but not gavage, could lead to the suppression of anti-Ova IgG1.

Our group has previously described that continuous administration of the antigen is an optimal protocol for oral tolerance induction when compared to antigen administration by gavage [49]. Even aged mice (70-week-old), usually refractory to oral tolerance by gavage, can be rendered tolerant by continuous feeding of the antigen [50]. We have also shown, in a previous study using mice with ethanol-induced colitis, that oral tolerance induction to specific serum IgG1 production was impaired in gavage-fed mice [51]. As already reported, IgG1 is the most resistant immunological parameter to suppression by mucosal administration of proteins. Nasal administration of antigen does not suppress specific anaphylactic serum IgG1, whereas continuous feeding inhibits it very efficiently [52]. In this study, we confirmed these previous data showing that continuous feeding but not gavage was able to render diseased IL-10-deficient mice tolerant to ovalbumin when specific IgG1 antibodies were measured. Therefore, IL-10 deficiency and severe inflammation in the gut mucosa seemed to decrease but not abolish susceptibility to oral tolerance induction. This result represents a key element in designing novel therapeutic approaches for inflammatory bowel diseases. Since these pathological conditions are generated by inflammation, the suppressive modulatory properties of tolerance procedures obtained by oral administration of disease-related proteins would be an alternative. Oral tolerance to target antigens has been successfully tested in many models of other inflammatory illnesses such as autoimmune and allergic diseases [6]. Our present data indicates that even in the presence of severe colitis and enteritis, oral tolerance approaches could be used as an alternative therapeutic tool for inflammatory bowel diseases. Nevertheless, optimal protocols of oral administration, such as continuous feeding, would be a requirement for the use of such strategies.

In conclusion, we demonstrated that IL-10-deficient mice have a progressive and spontaneous inflammation with marked alterations in the immune system. The absence of an important anti-inflammatory cytokine such as IL-10 leads to a dramatic defect of immunoregulation, with reduced regulatory T lymphocytes in the gut mucosa, alterations in immunoglobulins isotypes production and in the frequency of B1 cells. Importantly, we also showed that although several mechanisms of immunoregulation were compromised in IL-10-deficient mice, oral tolerance induction could still be induced. Our study also provides data showing that some immunoregulatory mechanisms were still preserved and oral tolerance could be induced even during overt inflammation.
